# Supplementary figures and images for: Recovery from heat shock requires the microRNA pathway in Caenorhabditis elegans
Source: PLoS Genet. 2021 Aug 5;17(8):e1009734. doi: 10.1371/journal.pgen.1009734 (PMC8370650; doi:10.1371/journal.pgen.1009734)

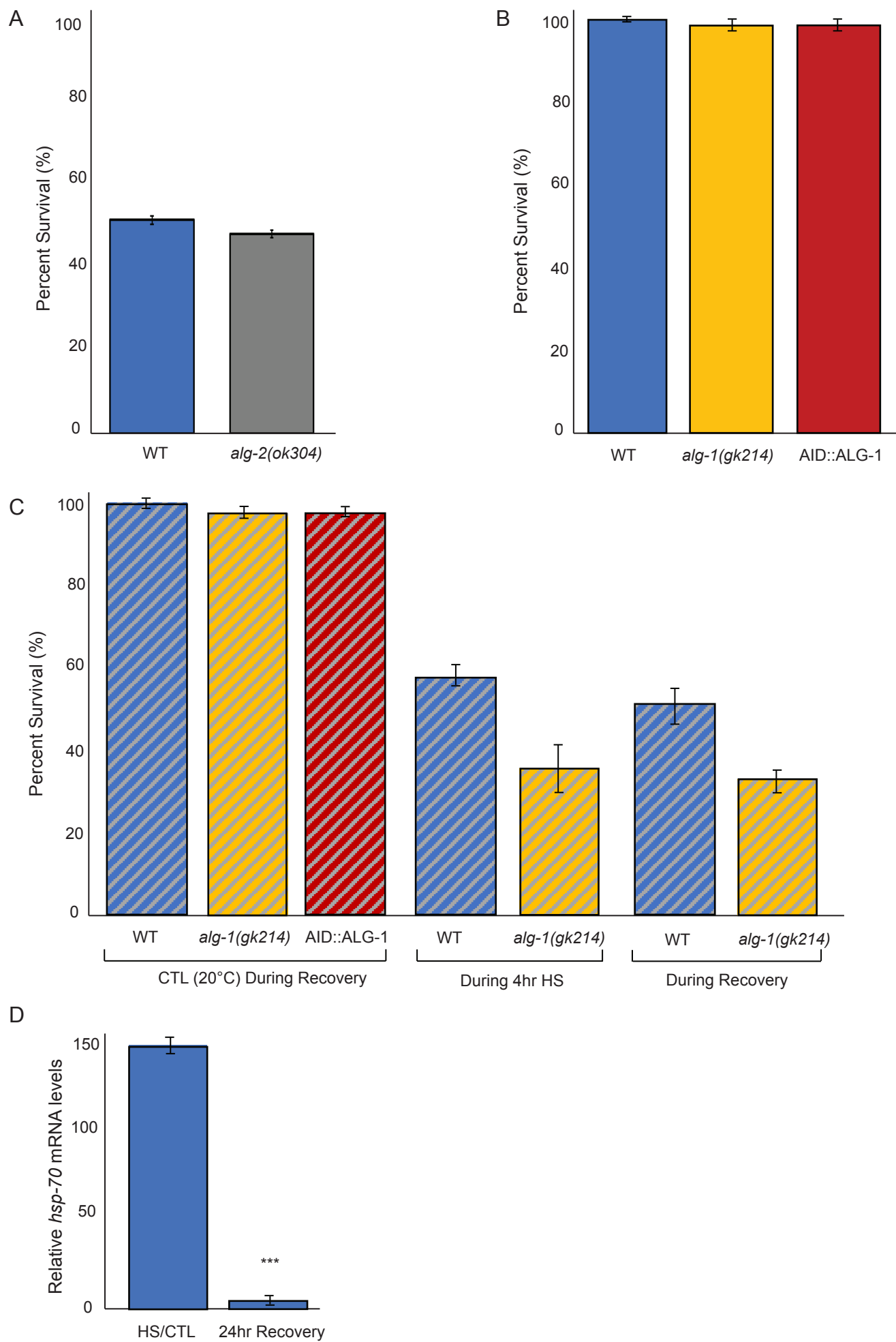

Supplement: S1 Fig — A. Percent survival of WT, alg-1(gk214), and AID::ALG-1 animals grown at 20°C for 72 hrs. Three biological replicates were performed with at least 50 worms per strain. Error bars represent SEM. B. Percent survival of synchronized WT and alg-2(ok304) animals grown at 20°C for 44 hrs before heat shock for 4 hrs at 35°C. Viability was scored after 24 hrs of recovery at 20°C. Three biological replicates were performed with at least 50 worms per strain. Error bars represent SEM. C. Percent survival of synchronized WT, alg-1(gk214), and AID::ALG-1 animals grown to stage L4 before being moved to auxin-containing media either for 24 hrs at 20°C, during 4 hrs HS, or during 24 hrs recovery after HS. Three biological replicates were performed with at least 50 worms per strain. Error bars represent SEM. D. RT-qPCR of hsp-70 mRNA levels in WT animals subjected to 3 hr HS and allowed to recover for 24hrs. All replicates were normalized to ama-1. Three biological replicates were assayed. Student’s t-tests were performed to determine significance relative to hsp-70 mRNA expression compared to HS (***P < 0.001). Error bars represent SEM. (PDF) [file pgen.1009734.s003.pdf]

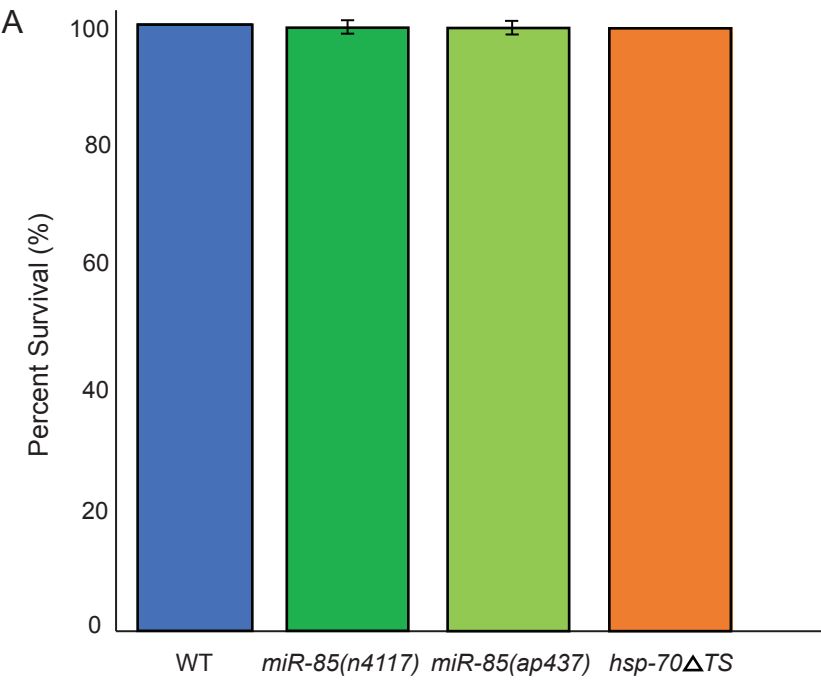

Supplement: S2 Fig — A. Percent survival of WT, miR-85(n4117), miR-85(ap437), and hsp-70ΔTS animals grown at 20°C for 72 hrs. Three blinded biological replicates were performed with at least 50 worms per strain. Error bars represent SEM. (PDF) [file pgen.1009734.s004.pdf]

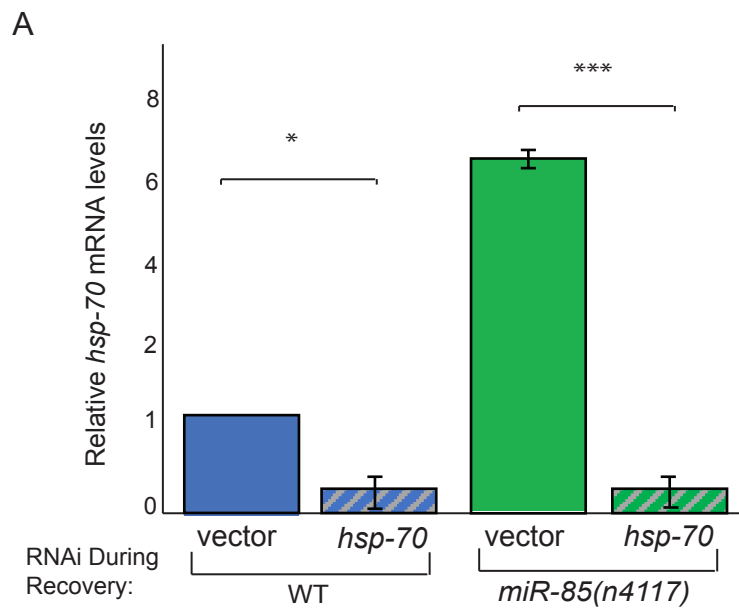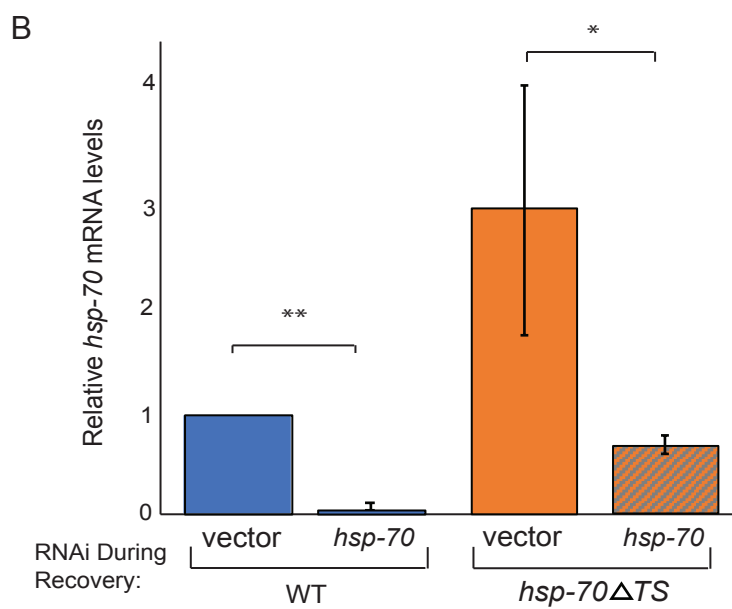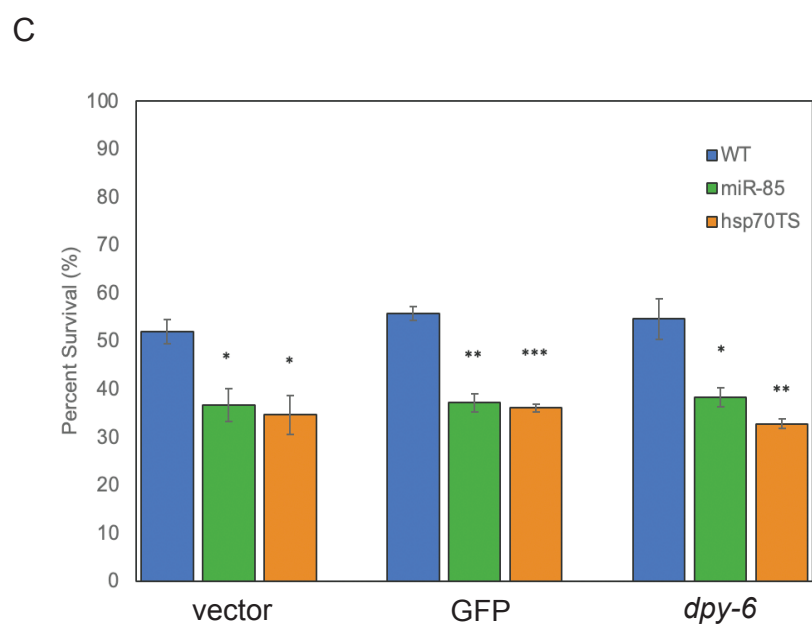

Supplement: S3 Fig — A. RT-qPCR of hsp-70 mRNA levels in WT and miR-85(n4117) animals subjected to HS and allowed to recover on vector RNAi or hsp-70 RNAi for 24 hrs. All replicates were normalized to ama-1 mRNA. Three biological replicates were assayed. Student’s t-tests were performed to determine significance relative to hsp-70 mRNA expression immediately after HS for each strain (***P < 0.001). Error bars represent SEM. B. RT-qPCR of hsp-70 mRNA levels in WT and hsp-70ΔTS animals subjected to HS and allowed to recover on vector RNAi or hsp-70 RNAi for 24 hrs. All replicates were normalized to ama-1 mRNA. Three biological replicates were assayed. Student’s t-tests were performed to determine significance relative to hsp-70 mRNA expression immediately after HS for each strain (*P < 0.05, **P <0.01). Error bars represent SEM. C. Following 4 hr of HS at 35°C, WT, miR-85(n4117), and hsp-70ΔTS animals were subjected to GFP and dpy-6 RNAi and percent survival was determined after 24 hrs recovery at 20°C. Three blinded biological replicates were performed with at least 50 worms per strain, per condition. Student’s t-tests were performed to determine significance for each strain relative to survival on vector RNAi (*P < 0.05, **P < 0.01, ***P < 0.001). Error bars represent SEM. (PDF) [file pgen.1009734.s005.pdf]
