# Supplementary material for: Recovery from heat shock requires the microRNA pathway in Caenorhabditis elegans
Source: PLoS Genet. 2021 Aug 5;17(8):e1009734. doi: 10.1371/journal.pgen.1009734 (PMC8370650; doi:10.1371/journal.pgen.1009734)
Supplement: Attachment — Submitted filename: 2021-6-22 Pagliuso Rev comments FINAL.docx [file pgen.1009734.s006.docx]

*We thank the reviewers for the very positive overall comments and thoughtful suggestions. We appreciate the opportunity to submit the revised version, which has been improved based on the reviewers’ insights. Where possible, additional experiments, analysis, and textual changes have been added to address reviewer comments, as detailed in* ***bold****. We now include an additional Supplemental Table 2 with all numerical data as required by the data availability policy. We have also embedded the Figure Captions within the manuscript after their first mention, as advised for PLoS formatting.*

Reviewer's Responses to Questions

**Comments to the Authors:
Please note here if the review is uploaded as an attachment.**

Reviewer #1: Pagliuso and collaborators report here the involvement of the microRNA pathway in the recovery of the nematode C. elegans upon heat shock. More particularly, the authors show that the Argonaute ALG-1, and more particularly a specific microRNA, miR-85, is needed for this biological process. Genetic and molecular experiments also suggest that miR-85 is involved in the regulation of hsp-70, a gene that needs to be down-regulated for allowing recovery and survival of the animal after heat shock.

Overall, this is a very interesting study that shed light on a new gene regulatory pathway important for animal recovery upon heat shock that involved a specific microRNA. While the experiments presented here strongly support the contribution of ALG-1 and miR-85 in this process, it is still unclear how the microRNA pathway precisely contributes to heat shock recovery as the miR-85/hsp-70 regulatory axis appears not sufficient to fully explain this biological process. Therefore, it is important to perform the following experiments to help better define how miR-85 controls this stress response.

-In silico identification of putative miR-85 targets and the comparative RNA sequencing analysis infer the potential regulation by miR-85 of other key mRNAs, besides hsp-70, involved in stress response. Furthermore, the data presented in Figure 3 suggest that miR-85 is needed for the acute clearance of hsp-70 mRNA, but at a later time point, the down-regulation of hsp-70 does not seem to directly involve miR-85 as hsp-70 mRNA deleted for miR-85 binding sites decreased substantially 16hr after heat shock. As mir-85 mutant retains high hsp-70 level after heat shock, other mRNAs targeted by miR-85 likely contributed to its down-regulation. Therefore, it will be important to test the involvement of other potential miR-85 targets in this process as performed for hsp-70. A subset of genes to be tested can be easily identified from the ones found by comparative RNA sequencing.

**We agree that higher levels of *hsp-70* at the later time points (16, 24 hr) after HS recovery in *mir-85* compared to *hsp-70ΔTS* animals suggest that other mis-regulated genes in *miR-85* (but not *hsp-70ΔTS*) contribute to down-regulation of *hsp-70* mRNA (and note this in the Discussion). Despite this difference, though, the HS viability phenotypes are strikingly similar for these 2 strains, suggesting that the later decline of *hsp-70* in *hsp-70ΔTS* animals is either too late or not enough (it is still quite high compared to WT). Nonetheless, we explored the reviewer suggestion to find other genes involved in regulation of *hsp-70* in the *miR-85* mutants. The reviewer suggested identifying potential miR-85 targets from the comparative RNA sequencing but the only miR-85 target gene that is differentially expressed in our RNA sequencing under control temperatures is *hsp-70*. To clarify this finding and emphasize why we focused on *hsp-70,* we included a Venn-diagram in Fig. 2 depicting the overlap of miR-85 targets from TargetScan and those upregulated by RNA sequencing. So, we then considered the genes that are up-regulated by HS but found no miR-85 targets differentially expressed in WT versus *miR-85* (even *hsp-70* is at similar levels likely because of the strong transcriptional induction by HS). So, finally we asked if any of the genes induced by HS are predicted miR-85 targets, even though they do not show differential expression in *miR-85.* Besides *hsp-70* there are 3 genes: *cls-2*, *unc-23*, and *F10D7.3*. When we tested if RNAi against these candidates might rescue the reduced viability of *miR-85* or *hsp-70ΔTS* strains, we found that they gave inconsistent results in 3 independent replicates and generally reduced the viability of WT animals, making it impossible to conclude anything about rescue activity in the mutants. This is in stark contrast to *hsp-70* RNAi, which had no effect on WT and consistently increased the survival of the *miR-85* or *hsp-70ΔTS* strains after HS. As a further control to confirm the specific effect of *hsp-70* RNAi, we now include HS viability assays recovered on *GFP* or *dpy-6* as controls (new Supplemental Fig. 3C). Neither *GFP* or *dpy-6(RNAi)* rescued HS viability of *miR-85(n4117)* or *hsp-70*Δ*TS* animals, nor did they affect WT viability*.***

-To better compare the effects on hsp-70 mRNA level detected in mir-85 mutant and in animals expressing hsp-70 without miR-85 target sites during heat shock recovery, plotting the data on the same graph will be necessary. That will allow the readers to appreciate the magnitude of effects seen in both conditions and determine the importance of the direct miR-85 regulation of hsp-70.

**This is a great suggestion and we have updated Figure 3A to present the data as suggested. The text has been updated to reflect this change.**

-To better define the contribution of miR-85 during heat shock recovery, it will be warranted to monitor its level at the same time points where hsp-70 mRNA level has been surveyed (0, 4, 16 and 24h).

**To confirm that miR-85 is present after HS, we performed a Northern blot probing for miR-85 24 hours after heat shock and added this data to Fig 3D. Additionally, we have Taqman qRT-PCR data showing no difference in miR-85 levels after HS recovery at 2 or 16 hr. We prefer to just include the Northern showing the last time point, but the Taqman can also be added, if needed.**

Besides those crucial issues, the following also need to be corrected:

-P.16, first paragraph: Figure 3E and 3A should be referred to rather than Figure 4E and 4A

**This has been corrected.**

-Figure 1F: Please show the complete base-pairing of the miR-85 with the site 2 of hsp-70 3’UTR

**Figure 1F has been updated as requested.**

-P.6, line 112: Besides the paper listed, the fertility of alg-1(gk214) animals has directly been surveyed in Bukhari et al., Cell Research 2012 paper. Please add this citation.

**This citation has been added to the text.**

Reviewer #2: The manuscript titled “Recovery from Heat Shock Requires the MicroRNA Pathway in
Caenorhabditis elegans” by Pagliuso et al. shows that miRNA pathway and specifically miR-85 in C. elegans is required to regulate the expression of the conserved heat shock protein HSP-70 during recovery from heat shock. This is a very interesting manuscript, well written and well presented. One particularly important aspect of the manuscript is that the authors focus on the recovery from heat shock. While there is many research publications describing stress response, attention to recovery from stress and how this part of the physiology is regulated has been lacking. In addition, the manuscript identifies an important biological role for the miRNA pathway in recovery from stress. The authors provide multiple lines of evidence of miRNA regulation of HSP-70 during the recovery phase by using miRNA miR-85 deletions lines, removal of sequences targeted by miR-85 within the HSP-70 3ʹ UTR.

I recommend the publication of the manuscript at PLoS Genetics once the following issues are addressed either through additional data analysis or further clarification within the manuscript text.

1- It is not sufficiently clear why the authors focused on hsp-70 among all the other heat shock proteins to begin with. Have the authors investigated the response of other HS factors?

**The only miR-85 target gene that is differentially expressed in our RNA sequencing under control temperatures is *hsp-70*. To clarify this finding and emphasize why we focused on *hsp-70,* we included a Venn-diagram in Fig. 2 depicting the overlap of miR-85 targets from TargetScan and those upregulated by RNA sequencing. Furthermore, *hsp-70* was an exciting candidate for post-transcriptional regulation during HS recovery based on studies in Drosophila by the Lindquist and Morimoto labs, as well as others. We have not yet explored whether other HSPs depend on the miRNA pathway for return to control temperature levels after HS but think this is a very interesting idea to pursue in the future.**

2- Authors use TargetScanWorm to identify two predicted miR-85 binding sites. Did the authors identify other miRNA binding sites in hsp-70 UTR?

**Other poorly conserved sites exist. This information was added to the text:**

**“While additional miRNAs are predicted to target *hsp-70*, miR-85 was the only miRNA with a target site conserved in other nematodes.”**

3- Regarding the RNA-Seq experiment of miR-85 mutants, I couldn’t see a link to the deposited data in materials & methods. The authors should also include some QC data on their RNA-Seq, RNA RIN values if available, read counts of libraries, % mapped, %uniquely mapped, % multi-mapper, PCA analysis between samples and to RIN values. If any custom codes has been used these should be provided as well to enable independent validation of the data.

**The link was provided in the submission window that asks where the data may be found (which is easy to miss):**

**All RNA Sequencing data files are available from the GEO database**

**(https://www.ncbi.nlm.nih.gov/geo/query/acc.cgi?acc=GSE168073__;!!Mih3wA!VTJBM**

**Qz4Sl5gF6hiGg8GqgFSETUZS7vzI3WyXfhPDYrddepB385AacGhMNa6RV1M$ ) with**

**accession number GSE168073 and this secure token (unyvicschxotjqn) to allow review until this work is accepted for publication.**

**We will make the files publicly available upon acceptance.**

**RNA was assessed for quality before sequencing by RNA Tapestation. RIN values for samples ranged between 8.4-10.0 with an average RIN value of 9.6. Library sizes ranged from 28-43 million reads. The average % uniquely mapped reads was 84.44% (SD 0.06). We added greater details regarding our quality assessment as commonly reported in the Methods sections. If the reviewer feels it would be valuable, we can also include the following summary table to our supplemental information.**

**The following was added to the Methods section regarding the RNA sequencing experiment:**

**“cDNA sequencing libraries from three independent biological replicates were prepared from 1ug of total RNA (with RIN > 8.4) using the standard protocol from the Illumina Stranded TruSeq RNA library prep kit. Ribosomal RNA was removed prior to library preparation using RiboZero Gold (Illumina). cDNA libraries were sequenced on the Illumina High Seq 4000 (SR75). Libraries were at least 28 million reads per sample. Reads were aligned to the *C. elegans* genome WS235 using STAR and the average percent of uniquely mapped reads was 84.4% [66].”**

| **Sample** | **RIN** | **Library Size/Reads (million)** | **% Uniquely Mapped** | **% Multi-mapped** |
| --- | --- | --- | --- | --- |
| **WT CTL 1** | **8.6** | **34.02** | **85.44%** | **12.82%** |
| **WT CTL 2** | **9.7** | **38.83** | **86.55%** | **12.56%** |
| **WT CTL 3** | **8.4** | **43.41** | **71.12%** | **28.11%** |
| **WT HS 1** | **9.3** | **31.91** | **81.55%** | **15.6%** |
| **WT HS 2** | **9.9** | **39.25** | **80.21%** | **18.77%** |
| **WT HS 3** | **9.8** | **40.74** | **79.89%** | **18.58%** |
| **miR-85 CTL 1** | **9.9** | **43.92** | **89.98%** | **7.10%** |
| **miR-85 CTL 2** | **9.8** | **28.69** | **90.64%** | **7.31%** |
| **miR-85 CTL 3** | **10** | **31.40** | **91.07%** | **6.79%** |
| **miR-85 HS 1** | **10.0** | **29.80** | **86.03%** | **12.11%** |
| **miR-85 HS 2** | **9.1** | **29.35** | **86.47%** | **11.23%** |
| **miR-85 HS 3** | **10** | **31.58** | **84.37%** | **13.84%** |

4- Line 162, Do the authors see enrichment of predicted miR-85 targets among the up regulated genes in comparison to the down-regulated genes?

**Analysis of the down-regulated genes under control conditions in *miR-85(n4117)* compared to WT (N2) did not reveal any genes with predicted miR-85 target sites. The only gene that had a predicted target site in the up-regulated genes was *hsp-70.* To further clarify this, we included a Venn-diagram in Fig. 2C of miR-85 targets compared to the up-regulated PCGs in *miR-85(n4117)* animals.**

5- Line 164, Can the authors compare the GO term enrichment analysis between the up and down regulated genes to see if the enrichment in up-regulated genes is specific to this set? How these Go enrichments are calculated is not clarified in materials and methods.

**1) The enrichment for up- and down-regulated genes are different. We now make this apparent in the text:**

**“Additionally, stress response and transit peptide were the only two terms identified among the upregulated genes by GO enrichment analysis using DAVID [34] (Fig 2B). Although the enrichment of these terms was modest, this finding is consistent with a role for miR-85 in regulating stress response genes. Down-regulated genes were enriched for terms associated with collagen, nucleosome assembly and modification, and signaling.”**

**2) To clarify how the GO enrichment was calculated, we added the following to the Materials and Methods:**

**“Gene Ontology enrichment was determined using DAVID Gene Ontology Analysis of the PCGs determined to be significantly up- or down-regulated in *miR-85(n4117)* compared to WT by RNA sequencing.”**

6- Page 7, line 139 - Authors mention that upon HS hsp-70 mRNA is up-regulated over 100 fold. However, in Figures 1 & 2, hsp-70 mRNA levels are only 2 fold higher when miR-85 regulation doesn’t happen during recovery. Can the authors clarify these differences and discuss more clearly how this is sufficient for the protein expression changes and the phenotypes?

**The *hsp-70* mRNA is up-regulated over 100-fold in response to HS. In control (20°C) conditions, with no exposure to heat stress, loss of miR-85 results in around a 2-fold upregulation as shown in Figures 1 & 2. After HS, very high levels of *hsp-70,* which resemble those achieved by HS, are maintained in the loss of function *miR-85* animals. It seems that the 2-fold increase in *hsp-70* in *miR-85* mutants under non-HS conditions is tolerable (no obvious developmental or viability phenotypes in *miR-85* mutants), but the very high levels *hsp-70* from transcriptional induction during HS depend on miR-85 to return to near non-stress levels. We tried to represent this role for miR-85 in clearing excess *hsp-70* after high HS levels in Fig 4E, but are open to suggestions for making this model clearer.**

Reviewer #3: Summary:
In their manuscript "Recovery from Heat Shock Requires the MicroRNA Pathway in Caenorhabditis elegans", Pasquinelli and colleagues analyze the requirement of miRNAs in a robust recovery from heat shock. They report that alg-1 mutants have reduced survival and retain elevated Hsp70, and that direct regulation of hsp-70 by miR-85 is required during recovery from heat stress.

This is a short and focused study of a miRNA-target pair that has physiologically relevant effects, and exploits the clean genetic analysis that is now possible in the worm. The work is generally well-done and uses excellent in vivo reagents (AID-Alg1, miRNA mutants and 3'UTR site mutants). It follows up on a line of thinking in the miRNA field in general about miRNA control in stress response, but these genetic tests are superior to most prior works and so are a good fit for Plos Genetics. I am supportive of publication following addressing a few issues raised.


Key findings:
• alg-1 mutants, which are known to have are various defects, are sensitive to heat shock. They further use temporally controled AID system to show that Alg1 is required concomitant with heat shock, avoiding pre-existing defects.

• hsp70 mRNA is induced 100X by heat shock, and returns to baseline by 24hrs. It's proposed not only due to transcriptional regulation, but post-transcriptional, since hsp70 is up about 2 fold in AID::ALG-1. miR-85 is a direct regulator of hsp70, since ∆mir85 mutants and an hsp70∆miR-85 sites (∆TS) strain shown 2-fold elevation of hsp70. While miR-85 and and hsp70∆TS strains seem normal, they have defective recovery from HS. The ∆mir85 can be rescued by miR-85 transgene, and hsp70-RNAi can increase survival of mir-85 and hsp70∆TS after heat shock.


Major concerns:
• I am not sure how precisely the hsp70-RNAi can result in depletion of Hsp70 protein. The rescue of mir-85 and hsp70∆TS mutants by hsp70-RNAi is a nice demonstration of the genetic impact of elevated Hsp70 in these mutants. However, its surprising since Hsp70 is a central coordinator of heatshock response. I think it could be quite difficult to precisely get back to a normal Hsp70 level by RNAi in these mutants and thus I think HS response could be negatively impacted. I may miss it, but I didn't see Hsp70 timecourse Western in these conditions (Figure 4) to know its induction and downregulation in hsp70-RNAi+mutants.

**We used qRT-PCR to specifically monitor expression of the inducible *hsp-70 (C12C8.1)* because this is not possible with antibodies against HSP-70, which likely detect the *hsp-70* homologs *F44E5.4* and *F44E5.5*. This caveat of the Westerns was noted in the text:**

**“It should be noted that the antibody used to detect HSP-70 likely reacts with homologs of this protein not expected to be regulated by miR-85 as their 3’UTRs lack binding sites for this miRNA. Thus, the Western blot results are likely an underestimate of the accumulation of HSP-70 in *miR-85(n4117)* compared to WT animals during recovery from HS.”**

**With RT-qPCR we were able to design primers specific to the HS-induced *hsp-70* (*C12C8.2*) transcript to assess the level of knock down by RNAi (Supp Fig. 3). Additionally, we checked that the RNAi clone was highly specific to this transcript.**

**We agree that it will be interesting to follow protein levels and hope to do this by using CRISPR to tag the endogenous *hsp-70,* but we do not yet have this reagent.**

• This is a very short report, which is ok. However, as they mention, organisms will face many stressors during life ("oxidative stress, pathogen infection, starvation, heat stress" etc. Let us add COVID19 life...) I think its relevant to test at least another, straightforward, stress response of ∆mir-85/ hsp70∆TS mutants to know the specificity. This is important for this study, because the mir-85/hsp70∆TS mutants have apparently no detectable phenotype in normal conditions.

**For this study, we focused on HS because we were able to achieve a highly reproducible viability assay where differences between WT and mutants could be confidently assessed. This required the purchase of a dedicated incubator for heat shock experiments and many trials to develop a protocol where about 50% of WT survived (so we could possibly detect increased or decreased survival of mutant strains). We are very interested in testing the role of miR-85 and hsp-70 regulation in other stress pathways and did try to establish an assay (UV, DTT, Ethidium bromide, paraquat, high salt) but have not yet succeeded in getting consistent results even for WT. We were able to achieve fairly reproducible results in a starvation assay where *miR-85(n4117)* or *hsp-70***∆***TS* animals appeared to have slightly reduced viability compared to WT, but this was not significantly different (4 independent replicates; t-test for WT vs *mir-85* = 0.12 and WT vs *hsp-70****∆****TS* = 0.08):**

**We do not think this result adds anything to the paper and in fact are not even sure what to conclude about it. However, it can be added if the reviewer thinks it necessary.**

**Our prediction is that stresses that induce high levels of hsp-70 will be at least partially dependent on miR-85 for recovery. Thus, it will also be important to analyze hsp-70 levels under the stress conditions once we establish reproducible protocols. While very interesting, we believe that carefully investigating the role of miR-85 in stresses besides HS is beyond the scope of this paper.**

Minor comment:
• What about hs response of alg-2, or some genetic interaction of alg-1 and alg-2?

**We assessed the HS viability in *alg-2(ok304)* animals. Loss of ALG-2 did not appear to hinder the viability of animals in our HS assay. The results are now included in SFig1B. The text has been updated as follows:**

**“Loss of *alg-2*, the most closely related homolog to *alg-1* in *C. elegans* [24], did not affect survival in this HS assay (S1B Fig). “**

• "Down-regulation of hsp-70 after heat shock is important for survival". Is the presumed misexpression of Hsp70 in mir/3'UTR mutants required in all tissues to cause the defect, or is there any evidence for tissue-specific preference of regulation for survival. I think the detailed analysis can be a future study, but I wonder if they can comment for discussion eg of mir85 expression or hsp70 induction preferences, since I think this is an important point for considering the biology of this regulation.

**This is a very important question for future study and will require new tools and methodologies to determine the expression and regulatory domains of miR-85 and *hsp-70*. While *hsp-70* is expected to be expressed everywhere, to our knowledge this has not been assessed for a tagged version of the gene that has its natural 3’UTR. We plan to generate such a strain but recognize that it has been historically challenging to tag hsp-70 and maintain function. While a promoter fusion for miR-85 was reported to show GFP expression in the uterus, spermatheca, gonadal sheath and rectal glands (Martinez et al., 2008), it is unclear where mature miR-85 is expressed. We are excited to try the mime-seq protocol developed in the Cochella lab to biochemically probe for mature miRNAs in specific tissues (Alberti et al., 2018) and expect that will better define where miR-85 potentially acts to regulate hsp-70. We are also working on a 3’UTR reporter to address this question but are far from having strains in hand.**

Reviewer #4: In this manuscript, Pagliuso and colleagues report on the involvement of microRNA miR85 in the heat-shock recovery (HSR) pathway in C. elegans. Leveraging CRISPR-Cas9 genome engineering and an acute protein degradation method (Auxin Induced Degradation), they show that deletion of ALG-1, miR85, or miR85 binding sites in hsp-70 mRNA are deleterious to the viability of the animal following heat-shock. They identify differentially regulated mRNAs in the miR85 deletion, and they show that loss of miR85, or deletion of miR85-binding sites in hsp-70 mRNA 3’UTR lead to persistence of hsp-70 mRNA and HSP-70 protein in HSR. Lastly, they show that hsp-70 RNAi in HSR rescues the viability phenotype associated with deletion of miR85 or its binding sites.

Overall, this is a sharply focused manuscript with well-controlled experiments and convincing results. The authors unveil the novel linkage of a microRNA and HSR, and convincingly demonstrate the physiological importance of miRNA-mediated repression and decay of the hsp-70 mRNA in this process. The manuscript also features elegant experimental designs including a clever use of AID, and CRISPR edition of 3’UTR sites, which phenocopies miR85 deletion and the HSR viability defects of alg-1 alleles. Considering the importance of HS and the limited prior knowledge on HSR molecular mechanisms, the manuscript should be appealing to a broad readership.

As it is often the case with briefer but exciting papers, the presented results raise several important questions that are left unanswered. Questions such as the reasons for the HSR viability defects resulting from hsp-70 persistence, the nature and mechanism of regulation of the miRNA function during HS remain open, are thoughtfully speculated upon in the Discussion section.

To this reviewer, the most surprising but under-developed piece of data in the manuscript is the observation that hsp-70 mRNA is under limited control by miR85 under normal (non-HS) conditions -hsp-70 mRNA is virtually not de-repressed in hsp-70 delta TS in Figure 3-, and that in spite of not being over-expressed upon HS, miR85 appears to become extremely efficient in the clearance of a strongly induced hsp-70 mRNA. While we recognize that this may not be within the current scope of the manuscript, we suggest two possible explanations that would be worth considering:

1- Is hsp-70 mRNA 3’UTR the same at basal level, and upon HS induction? Alternative polyadenylation sites can lead to dramatically different miRNA responses, and APA usage can be affected by transcription speed and activity. At the very least, the authors could consider performing a 3’RACE on hsp-70 mRNA at basal (non-HS) and upon HS and confirm that the 3’UTR really is what they expect it to be.

**This is a very interesting idea we had not previously considered. We attempted to perform 3’RACE to assess the 3’UTR of *hsp-70* during control and HS conditions but, after multiple attempts, were unable to successfully amplify a specific product and further work to develop specific primers will be necessary. To help assess the possibility of APA usage in HS, we compared the reads captured from RNA sequencing in control and HS of *hsp-70* during heat shock. We did not identify evidence to suggest alternative polyadenylation. Below are UCSC browser track images from WT animals in CTL and HS conditions.**

3’

5’

2- Another possible mechanism for this is the reorganization of 3’UTR structures upon HS. One could imagine miR85 target site availability to change on hsp-70 mRNA upon ‘melting’ the 3’UTR structures. Can the authors detect candidate folding structures surrounding or overlapping the two neighbouring miR85 binding sites?

**This is an intriguing hypothesis and warrants further investigation. We assessed the 3’UTR sequence of *hsp-70* for RNA secondary structure and included the findings below. It is striking that the 3’UTR can potentially fold back on itself like this, which would seem to obscure the miR-85 binding sites. Higher temperature could help open the stem, exposing the sites. It will be interesting to make reporters with the hsp-70 3’UTR and include mutants that test the importance of this potential structure.**

Citation: Lorenz, R. and Bernhart, S.H. and Höner zu Siederdissen, C. and Tafer, H. and Flamm, C. and Stadler, P.F. and Hofacker, I.L. "ViennaRNA Package 2.0", Algorithms for Molecular Biology, 6:1 page(s): 26, 2011

ACTTTGTA and TTTGTA (ACUUUGUA and UUUGUA) are the miR-85 seed sites

Minor comments:

Figure 3C is currently under-developed to an extent that it is next to useless. A more extensive description of the deg, even if only a few in this figure, would improve the value of this interesting experiment.

**To address this comment, we added labels for genes of interest to provide further information about the small subset of misregulated genes. As well, the complete list of up- and down-regulated genes from our RNA sequencing data can be found in Supplemental Table 1.**

This is the case for Figure 2A as well, where the authors could include an overlap of the OE mRNAs with predicted miR-85 binding sites beyond hsp-70 mRNA would add value to the manuscript.\

**To address this comment, we added a Venn-diagram to Fig. 2 showing the overlap of OE mRNAs with predicted miR-85 target sites. *Hsp-70* was the only mRNA shared. We hope this diagram helps clarify why we focused our study on *hsp-70.***

A consideration of the expression domain of miR85 would be valuable as well (where is it expressed? All cells in C. e. Involve miR85 in hsp70?).

**This is a very important question for future study and will require new tools and methodologies to determine the expression and regulatory domains of miR-85 and *hsp-70*. While *hsp-70* is expected to be expressed everywhere, to our knowledge this has not been assessed for a tagged version of the gene that has its natural 3’UTR. We plan to generate such a strain but recognize that it has been historically challenging to tag hsp-70 and maintain function. While a promoter fusion for miR-85 was reported to show GFP expression in the uterus, spermatheca, gonadal sheath and rectal glands (Martinez et al., 2008), it is unclear where mature miR-85 is expressed. We are excited to try the mime-seq protocol developed in the Cochella lab to biochemically probe for mature miRNAs in specific tissues (Alberti et al., 2018) and expect that will better define where miR-85 potentially acts to regulate hsp-70. We are also working on a 3’UTR reporter to address this question but are far from having strains in hand.**

**Have all data underlying the figures and results presented in the manuscript been provided?**
Large-scale datasets should be made available via a public repository as described in the *PLOS Genetics* [data availability policy](https://urldefense.com/v3/__http:/journals.plos.org/plosgenetics/s/data-availability__;!!Mih3wA!QWi90p2vZna4uORnxcYNOu8llcVipmK20AyZOpazXtD110tnQayiPdW49EM-Z5eqvuo$), and numerical data that underlies graphs or summary statistics should be provided in spreadsheet form as supporting information.

Reviewer #1: None

Reviewer #2: **No:** I couldn't locate the dataset for RNA-Seq experiments.

**The link was provided in the submission window that asks where the data may be found (which is easy to miss):**

**All RNA Sequencing data files are available from the GEO database**

**(https://www.ncbi.nlm.nih.gov/geo/query/acc.cgi?acc=GSE168073__;!!Mih3wA!VTJBM**

**Qz4Sl5gF6hiGg8GqgFSETUZS7vzI3WyXfhPDYrddepB385AacGhMNa6RV1M$ ) with**

**accession number GSE168073 and this secure token (unyvicschxotjqn) to allow review until this work is accepted for publication.**

**We will make the files publicly available upon acceptance.**

Reviewer #3: Yes

Reviewer #4: Yes

PLOS authors have the option to publish the peer review history of their article ([what does this mean?](https://urldefense.com/v3/__https:/journals.plos.org/plosgenetics/s/editorial-and-peer-review-process*loc-peer-review-history__;Iw!!Mih3wA!QWi90p2vZna4uORnxcYNOu8llcVipmK20AyZOpazXtD110tnQayiPdW49EM-KIm7Ulw$)). If published, this will include your full peer review and any attached files.


If you choose “no”, your identity will remain anonymous but your review may still be made public.


**Do you want your identity to be public for this peer review?** For information about this choice, including consent withdrawal, please see our [Privacy Policy](https://urldefense.com/v3/__https:/www.plos.org/privacy-policy__;!!Mih3wA!QWi90p2vZna4uORnxcYNOu8llcVipmK20AyZOpazXtD110tnQayiPdW49EM-qtoquh4$).

Reviewer #1: No

Reviewer #2: No

Reviewer #3: No

Reviewer #4: No
